# A systems pharmacology-based *in vivo* study elucidating the mechanism of Wengxian granules against avian salmonellosis

**DOI:** 10.3389/fvets.2026.1790708

**Published:** 2026-03-18

**Authors:** Siting Pu, Lihui Zhang, Hongzao Yang, Guangming Wang, Tingting Liu, Hongwei Chen, Wei Wei

**Affiliations:** 1 College of Veterinary Medicine, Southwest University, Chongqing, China; 2 School of Pharmaceutical Sciences, Tongren Polytechnic University, Guizhou, China

**Keywords:** immunity, molecular docking, network pharmacology, pharmacological mechanism, *Salmonella*

## Abstract

**Introduction:**

Avian salmonellosis is a major bacterial disease affecting the poultry industry. With increasing restrictions on antibiotic use in animal husbandry, alternative strategies for disease prevention are urgently required. Traditional Chinese medicine, characterized by a low risk of antimicrobial resistance, represents a promising alternative for sustainable livestock production.

**Methods:**

First of all, Pulsatillae radix‌ and six additional medicinal herbs were combined to formulate Wengxian granules, and their protective effects against avian salmonellosis were assessed. Then a systems pharmacology-based method integrating main active components screening, target prediction, network analyses, GO and KEGG analyses were used for the systematic deciphering of the mechanism of Wengxian granules in avian salmonellosis. Finally, based on systems pharmacology results, molecular docking was performed using AutoDock software.

**Results:**

Following Salmonella infection, the model group exhibited increased white and red blood cell counts and a reduced lymphocyte ratio compared with the control group. Severe detachment of the cecal mucosal layer was observed in the model group. Treatment with Wengxian granules significantly reversed these alterations by decreasing white and red blood cell counts and restoring the lymphocyte ratio, and protecting the integrity of the cecal mucosa and lamina propria. Immunological analysis showed that IgM, IgG and sIgA levels were elevated in the model group after infection, whereas Wengxian granule treatment reduced these levels. At the end of the experiment, immunoglobulin levels were significantly decreased in the model group compared with controls, whereas no significant differences were observed among the Wengxian-treated groups. Network pharmacology analysis identified 40 active compounds and 66 potential therapeutic targets associated with Wengxian granules. Kaempferol, β-sitosterol, quercetin, and hesperetin were identified as key bioactive components. KEGG pathway enrichment highlighted the Toll-like and NOD-like receptor signaling pathways as major pharmacological mechanisms involved. Molecular docking further confirmed stable interactions between core targets and active compounds.

**Discussion:**

Overall, these findings indicate that Wengxian granules exert protective effects against avian salmonellosis and provide mechanistic insights into their potential application as a non-antibiotic therapeutic strategy.

## Graphical abstract

**Figure:**
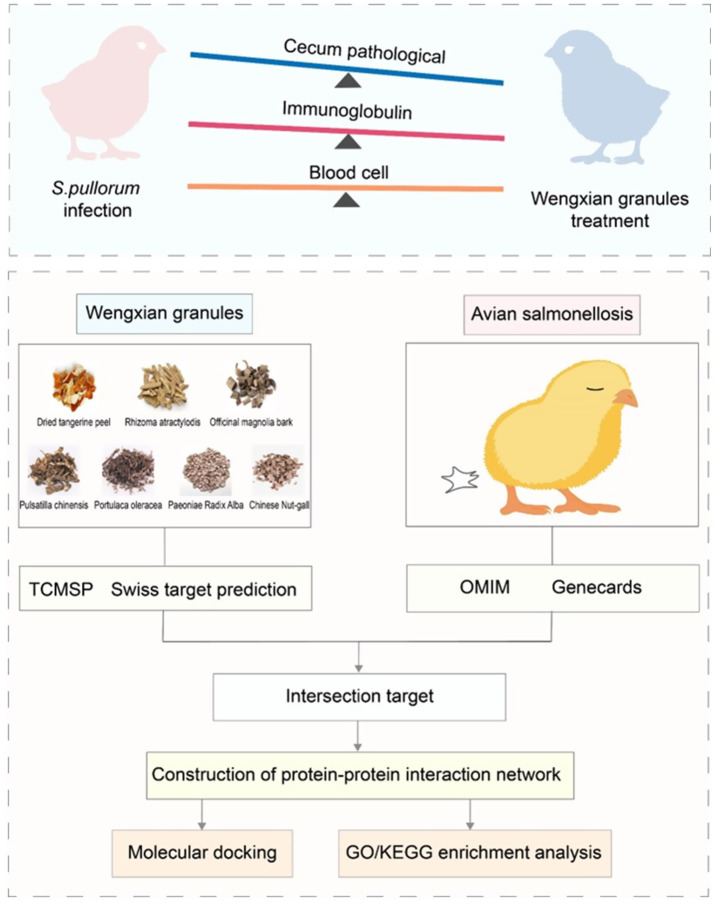

## 1 Introduction

Avian *Salmonella* infection, primarily including pullorum disease, avian typhoid, and avian paratyphoid, is a bacterial disease that poses a severe threat to global poultry production (1, 2). It is characterized by high mortality and growth retardation in chicks and substantial public health risks associated with vertical transmission and contamination of the food chain (3–5). *Salmonella pullorum* (*S. pullorum*) is the principal etiological agent of pullorum disease, which predominantly affects chicks aged 1–2 weeks and typically manifests as acute septicemia with high mortality (6). In adult birds, infections are often subclinical or chronic, leading to reduced egg production, decreased hatchability, and lower chick survival rates, causing considerable economic losses to the poultry industry (7).

Following oral infection with *S. pullorum*, bacterial lipopolysaccharide (LPS) is recognized by host pattern recognition receptors, triggering early anti-infective immune responses predominantly mediated by cellular immunity (8). During this process, polymorphonuclear leukocytes, cells of the mononuclear phagocyte system, circulating lymphocytes, and erythrocytes with immunoadsorption and immunomodulatory functions, collectively participate in pathogen clearance from the circulation (9). The antigenic structure of *Salmonella* is complex and consists mainly of somatic O antigens, flagellar H antigens, virulence-associated Vi antigens, and fimbrial antigens. Immunoprofiling studies have demonstrated that H antigens primarily induce IgG-dominated antibody responses, whereas O antigens preferentially elicit IgM responses (10). During mucosal invasion, *Salmonella* can stimulate B-cell differentiation within the intestinal lamina propria into plasma cells that produce secretory IgA (sIgA). Through immune exclusion, sIgA inhibits bacterial adhesion and internalization, serving as a key effector molecule in mucosal immunity against intestinal colonization and invasion (11–13).

Currently, the clinical prevention and control of avian salmonellosis still relies heavily on antibiotics. However, long-term antibiotic use not only increases production costs but also promotes the emergence and dissemination of drug-resistant strains, substantially compromising therapeutic efficacy (14). Owing to their natural origin, diverse pharmacological activities, low propensity to induce resistance, and minimal residue risks, traditional Chinese medicines have gradually attracted attention as promising alternatives to antibiotics. Accumulating evidence indicates that traditional Chinese medicines exert comprehensive preventive and therapeutic effects through direct antibacterial activity, modulation of gut microbiota, and enhancement of host immune function (15–17).

Based on the theory of syndrome differentiation and treatment in traditional Chinese medicine, avian salmonellosis is classified as damp-heat dysentery, and the therapeutic principle of “clearing heat and drying dampness, cooling blood, and detoxifying” has been proposed. In accordance with the holistic concept of traditional Chinese medicine and prescription composition principles, *Pulsatillae radix‌*, *Paeoniae Radix* Alba, *Portulaca oleracea*, *Galla chinensis*, *Magnolia officinalis*, *Atractylodis macrocephalae* Rhizoma*‌*, and *Pericarpium Citri* Reticulatae were combined to formulate the Chinese herbal compound Wengxian granules. Preliminary experiments have confirmed that Wengxian granules have a good antibacterial effect on *S. pullorum* (18). In order to further apply them in clinical practice, we investigated the effects of Wengxian granules on blood physiological indicators and immunoglobulin in chicks infected with *S. pullorum*, providing a basis for clinical medication. The formulation was incorporated into feed at predetermined ratios, and samples were collected at designated experimental time points. By integrating *in vivo* experiments with network pharmacology and molecular docking analyses, the preventive and therapeutic effects of Wengxian granules against pullorum disease in chicks, as well as their underlying mechanisms, were systematically investigated, thereby providing a potential new strategy for the clinical prevention and treatment of avian salmonellosis.

## 2 Materials and methods

### 2.1 Chemical and reagents

Wengxian granules were developed by the Traditional Chinese Medicine Innovation Laboratory, Veterinary Science and Engineering Research Center of Southwest University. Jililing powder was provided by Guangdong Tianbao Biopharmaceutical Co., Ltd. Chicken immunoglobulin G (IgG), immunoglobulin M (IgM), and secretory immunoglobulin A (sIgA) enzyme-linked immunosorbent assay (ELISA) kits were purchased from Shanghai Yaoyun Biotechnology Co., Ltd. A clinical isolate of *Salmonella pullorum* was obtained from the Microbiology Laboratory, Rongchang Campus of Southwest University, and the standard strain of *S. pullorum* was supplied by Hangzhou Baosai Biotechnology Co., Ltd. All other reagents used in the experiments were of analytical grade.

### 2.2 Animal experiment

A total of 120 one-day-old chicks were randomly allocated into six groups (*n* = 20 per group), including a control group, a model group, high-, medium-, and low-dose Wengxian granule groups, and a positive drug group. Prior to bacterial challenge, chicks in the control and model groups received normal saline, whereas those in the Wengxian granule groups were administered the formulation at doses of 0.5, 0.3, and 0.1 g per chick, respectively. The positive drug group received Jililing powder at a dose of 0.5 g per chick. Jililing powder is derived from the *Veterinary Pharmacopoeia of the People’s Republic of China II*, and Jililing powder is mainly used for treating pullorum disease (19). As reported in the literature (20), Jililing powder has a therapeutic effect on pullorum disease. All treatments were administered twice daily for 14 consecutive days. After continuous administration for 7 d, heart blood and cecal samples were collected from the selected chicks for baseline analysis. Subsequently, chicks in the model group and all treatment groups were challenged via intraperitoneal injection with a bacterial suspension containing 4.9 × 10^7^ CFU of *S. pullorum*. One day after infection, heart blood and cecal samples were collected from the selected chicks. Thereafter, all treatment groups continued to receive their respective formulations for an additional 7 days, after which heart blood and cecal tissues were collected for further analyses. Animal studies were approved by the Southwest University Animal Care and Use Committee (#IACUC-20260106-04). Experiments were conducted according to the NIH’s guidelines for the care and use of laboratory animals.

### 2.3 Histopathological examination

The mid-sections of the cecum were collected from the chicks and fixed in 4% paraformaldehyde for 1 week. After dehydration, tissues were embedded in paraffin and sectioned for histological analysis. The paraffin sections were stained with hematoxylin and eosin (H&E), and histopathological changes in the cecal tissues were examined under a light microscope (Nikon, ECLIPSE C1).

### 2.4 Cell counting

Chicks were randomly selected from each experimental group before the experiment, after infection, and at the end of the experiment, respectively. Heart blood samples were collected from the selected chicks and diluted 200 fold prior to analysis. A drop of the diluted blood sample was placed at the edge of the junction between the counting chamber and the coverslip and allowed to diffuse naturally into the chamber. After standing for 2–3 min, microscopic examination was performed. White blood cells (WBCs) were counted in the four corner large squares of the counting chamber under a low-power microscope, whereas red blood cells (RBCs) were counted in the four corner medium squares and the central medium square within the central large square. Dried blood smears were prepared and stained using Wright’s staining method. After drying, the smears were examined using an oil immersion lens. The numbers of WBCs and lymphocytes were determined using the four-corner area method, and the lymphocyte ratio was subsequently calculated.

### 2.5 Determination the contents of IgG, IgM, and sIgA

Blood samples were collected from the chicks before the experiment, after infection, and at the end of the experiment. They were subsequently centrifuged to obtain serum for subsequent analysis. Meanwhile, cecal tissues were collected, homogenized, and centrifuged. The supernatants were harvested for further analysis. Serum IgG and IgM contents were measured using chicken IgG and IgM ELISA kits (Yaoyun Inc., Shanghai, China), respectively. The content of secretory IgA (sIgA) in cecal tissues was determined using a chicken sIgA ELISA kit (Yaoyun Inc., Shanghai, China) according to the manufacturer’s instructions.

### 2.6 Screening of main active components and targets of Wengxian granules

The chemical components of Wengxian granules were screened using the Traditional Chinese Medicine Systems Pharmacology Database and Analysis Platform (TCMSP), with “*Pulsatillae radix‌*,” “*Paeoniae Radix* Alba,” “*Portulaca oleracea*,” “*Galla chinensis*,” “*Magnolia officinalis*,” “*Atractylodis macrocephalae* Rhizoma*‌*,” and “*Pericarpium Citri* Reticulatae” as keywords (21). Oral bioavailability (OB) ≥ 30% and drug-likeness (DL) ≥ 0.18 were used as screening criteria to identify active ingredients and their corresponding target proteins. The corresponding targets of the active ingredients were further retrieved using the SwissTargetPrediction (22) and Uniprot databases (23). After target standardization and removal of duplicates, the results were summarized. A “drug–active ingredient–target” interaction network was constructed and visualized using Cytoscape version 3.9.1 software (24).

### 2.7 Screening of disease targets

Targets associated with avian salmonellosis were retrieved from the GeneCards (25) and OMIM databases (26) using the keyword “avian salmonellosis.” Gene names were standardized and deduplicated to establish a disease-related target dataset. Venny 2.1.0 (27) was used to identify the intersection between disease-related targets and the predicted targets of Wengxian granules.

### 2.8 Protein–protein interaction network screening

The intersecting targets of active ingredient-related targets and avian salmonellosis-related targets were imported into the STRING database (28) to construct a protein–protein interaction (PPI) network. The species was set as *Gallus gallus*, with a confidence score threshold of ≥ 0.04. The interaction data were downloaded and analyzed using Cytoscape version 3.9.1 to calculate node degree values and visualize the network. The cytoHubba plugin was used for topological and centrality analyses using the maximal clique centrality algorithm to identify key targets and subnetworks.

### 2.9 GO functional and KEGG pathway enrichment analysis

Intersecting targets were imported into the DAVID database (29) for Gene Ontology (GO) functional annotation and Kyoto Encyclopedia of Genes and Genomes (KEGG) pathway enrichment analyses. Pathways with a corrected *p* value < 0.005 were considered significantly enriched, and genes involved in these pathways were further analyzed through gene–pathway network construction.

### 2.10 Molecular docking

Core active components and key targets were subjected to molecular docking analysis. The three-dimensional structures of the active components were obtained from the PubChem database (30), and the crystal structures of key target proteins were downloaded from the RCSB PDB database (31). Prior to docking, hydrogen atoms were added, and charges were calculated for structural preprocessing. Semi-flexible molecular docking was performed using AutoDock software (32), and docking results were visualized using PyMOL (33).

### 2.11 Statistical analysis

All experimental data were analyzed using GraphPad Prism (version 7.04). Statistical significance was assessed using analysis of variance (ANOVA), and the results were presented as the mean ± standard deviation (SD). Differences were considered statistically significant at *p* < 0.05.

## 3 Results

### 3.1 Wengxian granules against avian *Salmonella* infection through cell-mediated immunity

**Figure 1:**
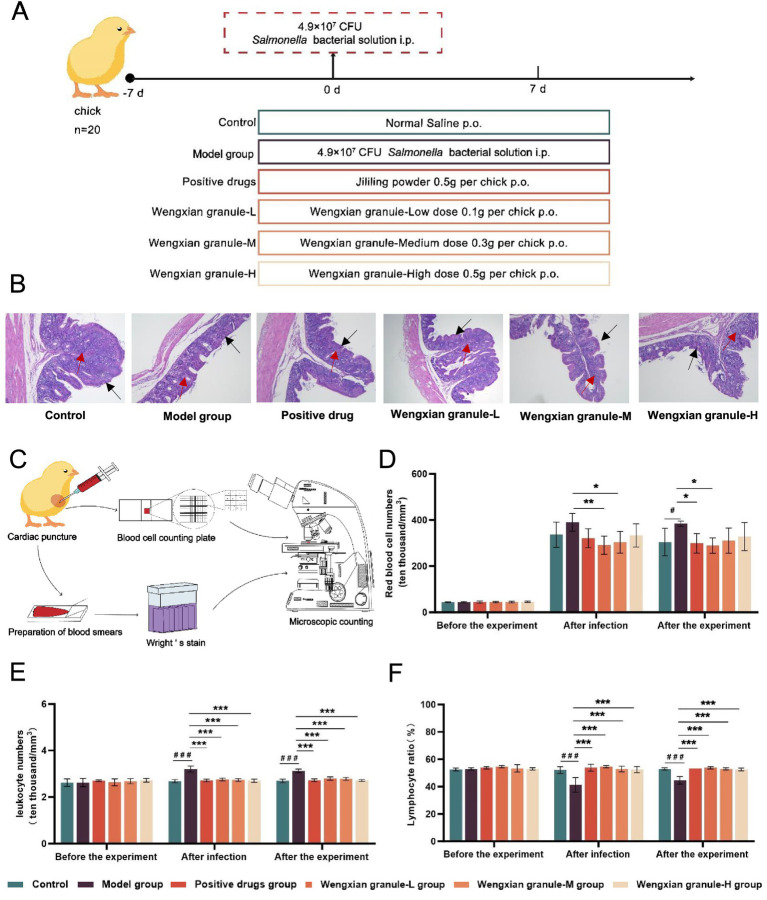
Wengxian granules relieve cecal damage through cellular immunity. **(A)** Schematic diagram of animal grouping and treatment. **(B)** Effect of Wengxian granules on the cecal tissue of chicks infected with *S. pullorum*. The black arrow represents the mucosal epithelium and the red arrow indicates the lamina propria. **(C)** After the experiment, we took the heart blood of chicks for cell counting. **(D)** Effect of Wengxian granules on the number of red blood cells in chicks infected with *S. pullorum*. Data are expressed as mean ± SD (*n* = 3). *p-*values are from one-way analysis of variance (ANOVA) followed by Tukey’s multiple comparison tests. **(E)** Effect of Wengxian granules on the number of white blood cells in chicks infected with *S. pullorum*. Data are expressed as mean ± SD (*n* = 3). *p*-values are from one-way analysis of variance (ANOVA) followed by Tukey’s multiple comparison tests. **(F)** Effect of Wengxian granules on the ratio of lymphocytes in chicks infected with *S. pullorum*. Data are expressed as mean ± SD (*n* = 3). *p*-values are from one-way analysis of variance (ANOVA) followed by Tukey’s multiple comparison tests. *p < 0.05, #*p* < 0.05, **p < 0.01, *** *p* < 0.001, ### *p* < 0.001.

A total of 120 chicks were randomly allocated to five experimental groups (20 chicks/group; Figure 1A). Pullorum disease is characterized by an acute septicemic course, with cecal lesions frequently observed in infected chicks. Histopathological examination demonstrated that the cecal mucosal epithelium and lamina propria in the control group remained intact, displaying well-defined structures and clear boundaries. In contrast, severe detachment of the cecal mucosal layer was observed in the model group. Treatment with high- and medium-dose Wengxian granules, as well as the positive drug, preserved the integrity of the cecal mucosa and lamina propria. In the low-dose Wengxian granule group, only mild detachment of the cecal mucosal layer was observed (Figure 1B). As documented in the literature (34), the specific scoring rules are tabulated in Supplementary Table S1. Sectioning and scoring were performed in a blinded manner by two independent observers.

Avian erythrocytes express complement receptors on their surfaces. When *Salmonella* is opsonized by the complement system, adhesion to erythrocytes can occur via these receptors, thereby facilitating bacterial transport to the liver and spleen for centralized clearance by phagocytic leukocytes. Heterophils play a critical role in host defense against pathogens through multiple mechanisms, including phagocytosis of foreign invaders, enhancement of chemotaxis and adhesion, degranulation, induction of respiratory burst, and production of cytokines and chemokines (35). Furthermore, lymphocytes secrete substantial amounts of interferon-gamma, which contributes to macrophage activation. In addition, cytotoxic lymphocytes can directly recognize and lyse host cells infected with *S. pullorum*. Based on these cellular immune responses, heart blood samples were collected from the chicks for peripheral blood cell counting (Figure 1C).

Before the experiment, no significant differences were detected among the groups in white blood cell (WBC) counts, lymphocyte levels, or red blood cell (RBC) counts. Following infection, the model group exhibited a significant increase in WBC count, accompanied by a reduction in lymphocyte percentage and an increase in RBC count compared with the blank control group. In contrast, all Wengxian granule-treated groups exhibited decreased WBC counts, increased lymphocyte percentages, and reduced RBC counts relative to the model group. After 7 days of continuous administration, the Wengxian granule-treated groups consistently maintained significantly lower WBC counts, higher lymphocyte percentages, and reduced RBC counts than the model group (*p < 0.05 and ^#^*p* < 0.05, **p < 0.01, *** *p* < 0.001 and ^###^ *p* < 0.001) (Figures 1D–F).

### 3.2 Wengxian granules against avian *Salmonella* infection through antibody-mediated immunity

In avian hosts, sIgA, IgM, and IgG constitute a comprehensive humoral defense system against *Salmonella* infection, functioning at local and systemic levels and providing protection from the early to later stages of infection. sIgA primarily acts at the intestinal mucosal barrier, where it limits bacterial colonization and invasion at entry sites (36). During the early phase of infection, IgM produced by plasma cells contributes to the control of systemic dissemination through complement activation and bacterial agglutination. As infection progresses, IgG generated in peripheral immune organs, such as spleen and bone marrow, exerts potent opsonization and neutralization effects and cooperates with phagocytes to facilitate clearance of systemic infections.

**Figure 2:**
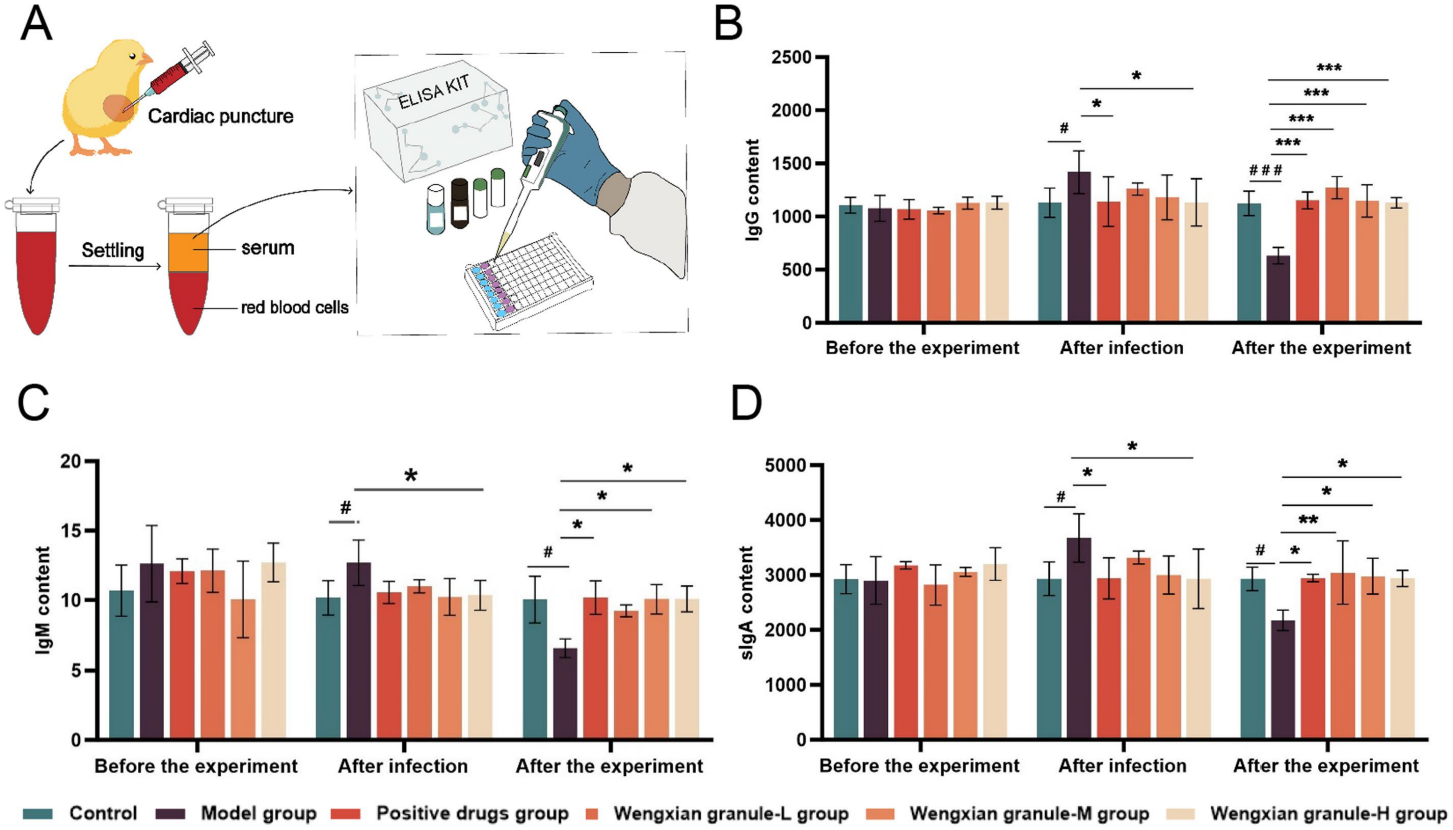
Effects of Wengxian granules on the levels of serum IgG, IgM, and Cecal sIgA in chicks infected with *S. pullorum*. **(A)** For each experimental group, chicks were randomly sacrificed at each time point (before the experiment, after infection, and after the experiment) to collect cardiac blood and cecal tissue samples for assay. **(B)** Effect of Wengxian granules on the IgG content in the blood of chicks infected with *S. pullorum*. Data are expressed as mean ± SD (*n* = 3). *p*-values are from one-way analysis of variance (ANOVA) followed by Tukey’s multiple comparison tests. **(C)** Effect of Wengxian granules on the IgM content in the blood of chicks infected with *S. pullorum*. Data are expressed as mean ± SD (*n* = 3). *p*-values are from one-way analysis of variance (ANOVA) followed by Tukey’s multiple comparison tests. **(D)** Effect of Wengxian granules on the sIgA content in the cecal tissue of chicks infected with *S. pullorum*. Data are expressed as mean ± SD (*n* = 3). *p*-values are from one-way analysis of variance (ANOVA) followed by Tukey’s multiple comparison tests.

To evaluate humoral immune responses, serum and cecal samples were collected from the chicks for immunoglobulin determination (Figure 2A). No significant differences in serum IgG levels were observed among the groups before the experiment or after infection (Figure 2B). Following infection, the serum IgG were significantly increased compared to those in the control group, positive drug group, and all Wengxian granule-treated groups (*p < 0.05 and ^#^*p* < 0.05). After the experiment, the serum IgG levels in the model group were significantly decreased compared with those in the control group, positive drug group, and high-, medium-, and low-dose Wengxian granule groups (***p < 0.001 and ^###^*p* < 0.001). Following infection, the serum IgM levels in the model group were significantly increased compared to those in the control group, positive drug group, and all Wengxian granule-treated groups (*p < 0.05 and ^#^*p* < 0.05) (Figure 2C). However, after the experiment, serum IgM levels in the model group were significantly lower than those observed in the other groups (*p < 0.05 and ^#^*p* < 0.05). In the early stages of infection, *Salmonella* antigens stimulated the body to produce IgG and IgM, resulting in a significant increase in IgG and IgM levels in the serum of the model group chicks. Wengxian granules inhibited the increase of IgG and IgM caused by *Salmonella* antigens. After the experiment, the IgG and IgM levels in the model group were lower than those in the control group, and *Salmonella* caused immune suppression in the chicks.

Before the experiment, no significant differences were detected in cecal sIgA levels among the groups. Following infection, the model group exhibited a significant increase in cecal sIgA content compared with the control group (^#^*p* < 0.05). By the end of the experiment, a highly significant decrease in cecal sIgA levels was observed in the model group compared to the control group (^#^*p* < 0.05). In contrast, no abnormal changes in cecal sIgA levels were detected in the Wengxian granule-treated groups (Figure 2D).

### 3.3 Screening active components and potential targets of Wengxian granules

**Figure 3:**
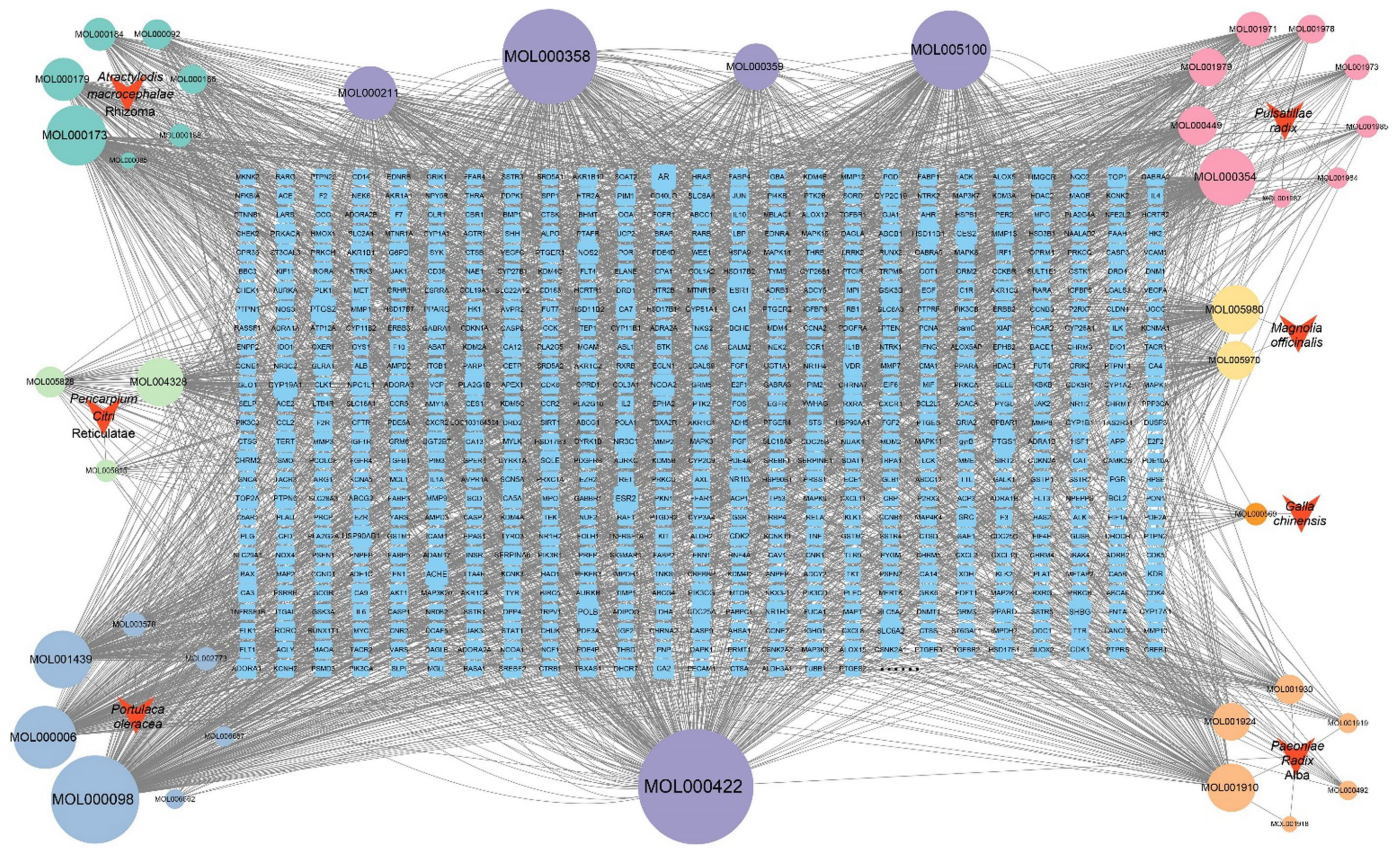
The active components of Wengxian granules were predicted, and the gene targets were enriched and analyzed to construct a network diagram of traditional Chinese medicine-active ingredient-target. Purple denotes components that are common to two or more herbal medicines, where the node size corresponds to the strength of the association.

Traditional Chinese medicine formulas are characterized by multi-target and multi-level mechanisms of action. Network pharmacology provides an effective approach for integrating large-scale information to identify potential active components and their corresponding targets (37). Network pharmacology analysis of the core herbal composition of Wengxian granules identified 40 active components. These included 10 components from *Portulaca oleracea*, 11 from *Pulsatillae radix‌*, 10 from *Paeoniae Radix* Alba, 7 from *Atractylodis macrocephalae* Rhizoma*‌*, 2 from *Magnolia officinalis*, and 1 from *Galla chinensis* (Supplementary Table S2). Among the identified components, kaempferol was shared by *Portulaca oleracea* and *Paeoniae Radix* Alba. *β*-sitosterol was common to *Paeoniae Radix* Alba, *Pulsatillae radix‌*, and *Portulaca oleracea*. Quercetin was derived from *Portulaca oleracea*, hesperetin was a common component of *Portulaca oleracea* and *Pericarpium Citri* Reticulatae. A total of 2,949 potential targets were identified, with contributions of 1,314 from *Portulaca oleracea*, 813 from *Pulsatillae radix‌*, 992 from *Paeoniae Radix* Alba, 382 from *Atractylodis macrocephalae* Rhizoma*‌*, 457 from *Pericarpium Citri* Reticulatae, 174 from *Magnolia officinalis*, and 21 from *Galla chinensis*. A total of 2,949 potential targets were identified, with contributions of 1,314 from *Portulaca oleracea*, 813 from *Pulsatillae radix‌*, 992 from *Paeoniae Radix Alba*, 382 from *Atractylodis macrocephalae* Rhizoma*‌*, 457 from *Pericarpium Citri Reticulatae*, 174 from *Magnolia officinalis*, and 21 from *Gallae Chinensis*. After merging and removing duplicates, 887 unique targets were retained. Using Cytoscape software, a “drug–active ingredient–target” network was constructed (Figure 3). The network comprised 933 nodes, including 6 traditional Chinese medicines, 40 active ingredients, 887 targets, and 3,461 edges. Network topology analysis identified the top 10 active ingredients with the highest degree values, which may represent the core active components involved in the treatment of avian salmonellosis (Supplementary Table S2).

### 3.4 Collection of avian salmonellosis targets and construction of protein–protein interaction network

**Figure 4:**
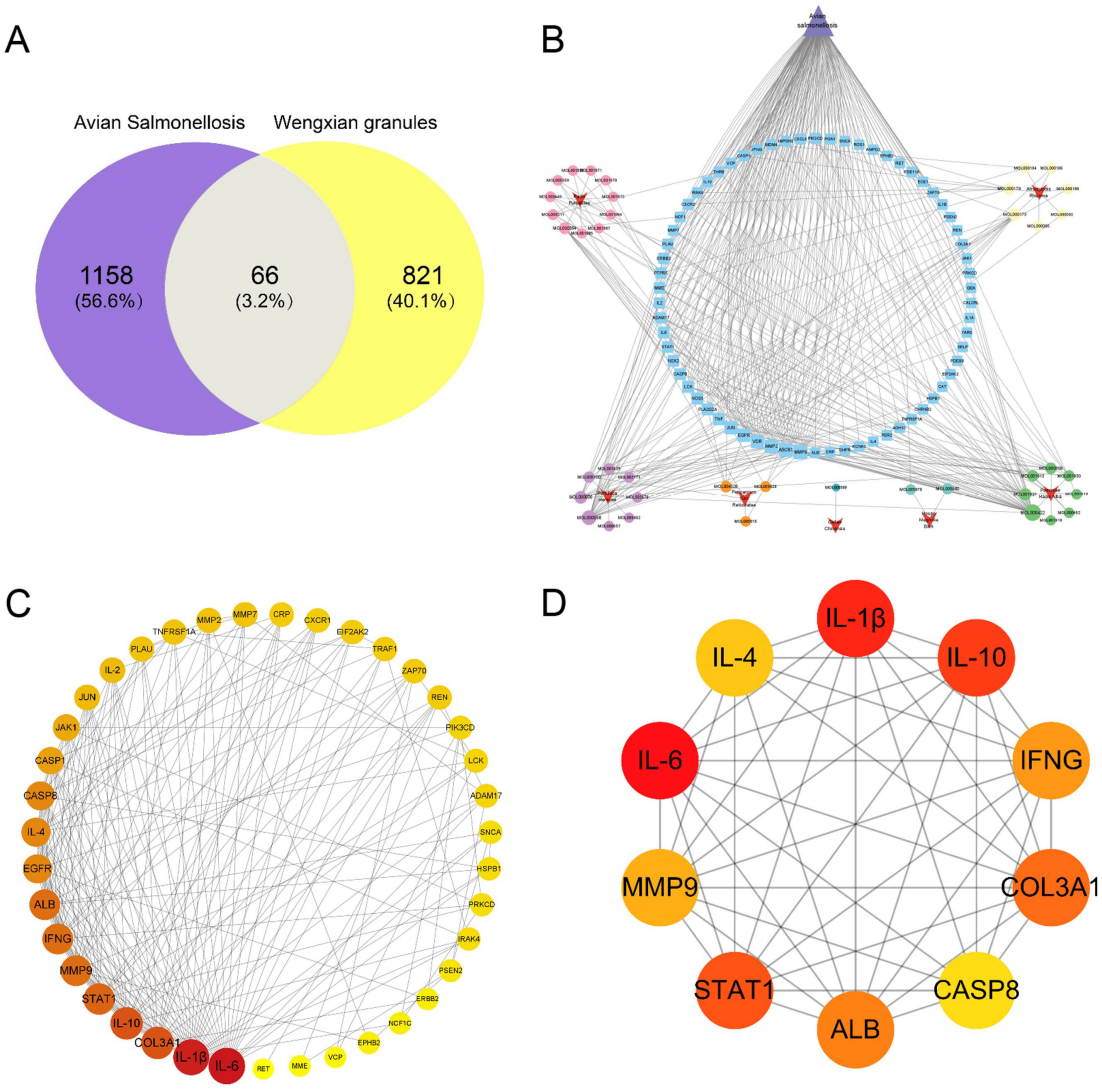
Target screening and establishment of PPI network. **(A)** Venn diagram illustrating the targets of Wengxian granules and avian salmonellosis. The purple section represents targets associated with avian salmonellosis, the yellow section represents targets associated with Wengxian granules, and the overlapping central area indicates the common targets of Wengxian granules and avian salmonellosis. **(B)** Network pharmacology analysis of active components and targets of Wengxian granules in the treatment of avian salmonellosis. **(C)** PPI network of Wengxian granules in the treatment of avian salmonellosis. The visualization is defined where node color saturation and size correspond to the magnitude of the degree centrality value. **(D)** Hub genes and subnetworks in the PPI network.

Targets associated with avian salmonellosis were retrieved from the GeneCards and OMIM databases. After merging and removing duplicates, a total of 1,224 disease-related targets were obtained. Venn analysis identified 66 overlapping targets between the predicted targets of Wengxian granules and avian salmonellosis–related targets (Figure 4A). Using Cytoscape software, visual networks of “Wengxian granules formulation–active ingredients–targets” and “avian salmonellosis-related targets” were constructed (Figure 4B). The 66 overlapping targets were subsequently imported into the STRING database for PPI analysis, with the species set as *Gallus gallus* and a confidence score threshold of 0.4. The resulting PPI network consisted of 39 nodes and 193 edges (Figure 4C). Based on the degree value analysis, the key targets of Wengxian granules in the treatment of avian salmonellosis were identified (Supplementary Table S3), among which IL-6, IL-1β, IL-10, and COL3A1 exhibited relatively high interaction frequencies. A core subnetwork was extracted using topological analysis with the cytoHubba plugin (Figure 4D).

### 3.5 GO functional and KEGG pathway enrichment analysis

**Figure 5:**
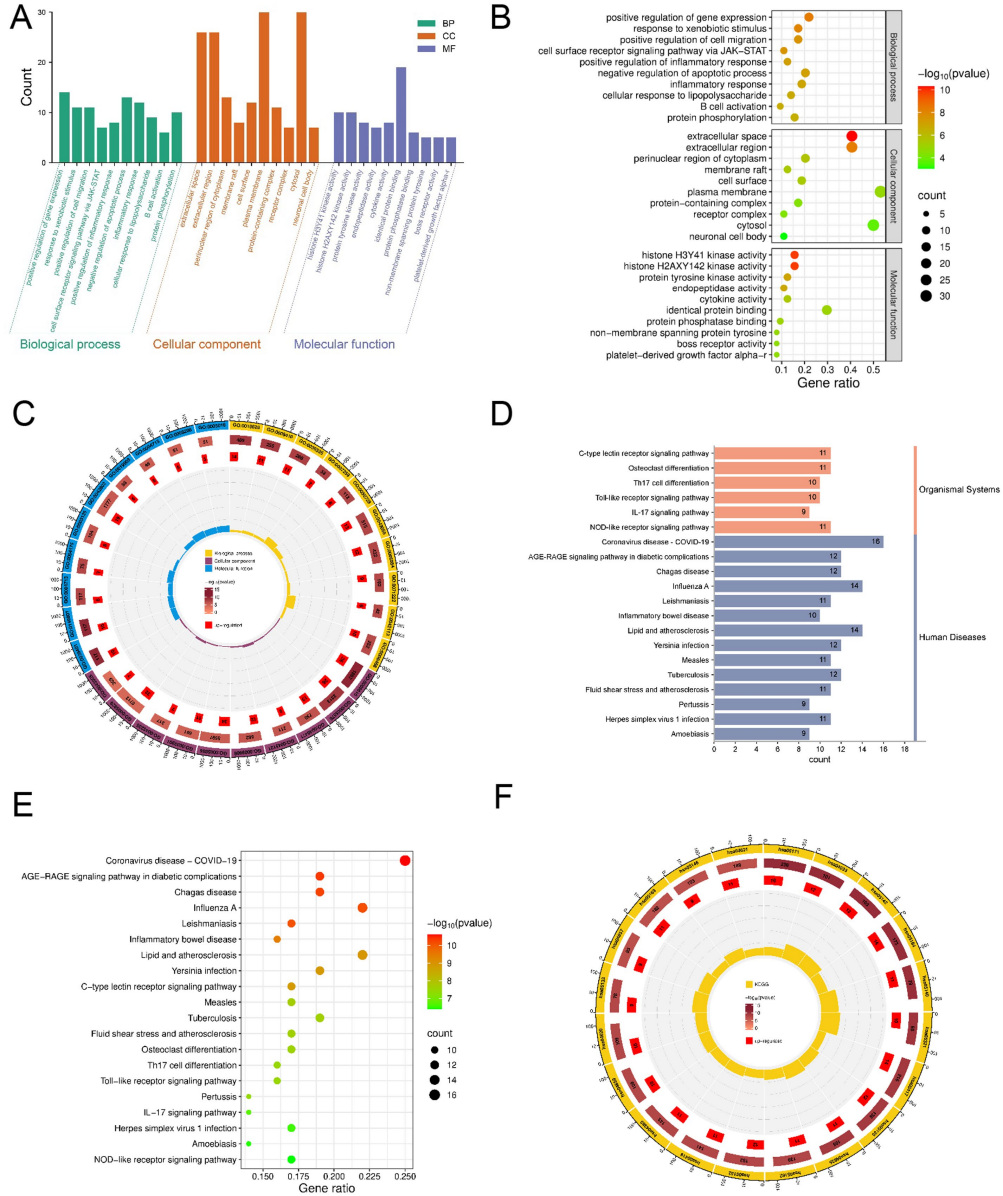
Enrichment analysis of GO biological process and KEGG pathways. **(A)** The top 10 items arranged in ascending order of *p* value were selected to generate a tripartite GO bar chart, including biological process (BP), cellular component (CC), and molecular function (MF). **(B)** GO analysis bubble chart. The size of the dots corresponds to the gene enrichment count, while the color represents the *p*-value. **(C)** GO analysis circle plot. From the outer to the inner ring, the successive layers represent: the first ring displays GO classifications, with terms of the same category color-coded identically; the second ring indicates the number of genes contained within each term, where color intensity reflects the *p* value; the third ring shows the number of up- or down-regulated genes in each term; and the fourth ring represents the rich factor. **(D)** The bar chart shows the top 20 pathways from the KEGG pathway enrichment analysis, with the length of each bar representing the number of genes. Longer bars indicate a greater number of genes and a higher level of enrichment. **(E)** Pathways enriched in the KEGG enrichment analysis related to the anti-avian salmonellosis mechanism of Wengxian granules were ranked by *p* value, and the top 20 pathways were visualized using a bubble plot. **(F)** KEGG analysis circle plot.

The 66 overlapping targets were subjected to GO functional annotation and KEGG pathway enrichment analysis using the DAVID database. GO analysis indicated that the biological processes associated with the targets of Wengxian granules in avian salmonellosis were mainly related to the positive regulation of gene expression, inflammatory responses, and negative regulation of apoptosis (Figure 5A). In terms of cellular components, the enriched targets were primarily associated with the extracellular region, perinuclear cytoplasm, and protein complexes. Molecular function analysis demonstrated enrichment in histone kinase activity, cytokine activity, and protein phosphatase binding. The top 10 GO terms for biological process, cellular component, and molecular function categories are presented in Figures 5B,C. KEGG pathway enrichment analysis revealed that the targets of Wengxian granules were mainly involved in signaling pathways related to Toll-like receptors and NOD-like receptors in the context of *Salmonella* infection (Figures 5D,E).

### 3.6 Molecular docking of active components from Wengxian granules with key target proteins of avian salmonellosis

**Figure 6:**
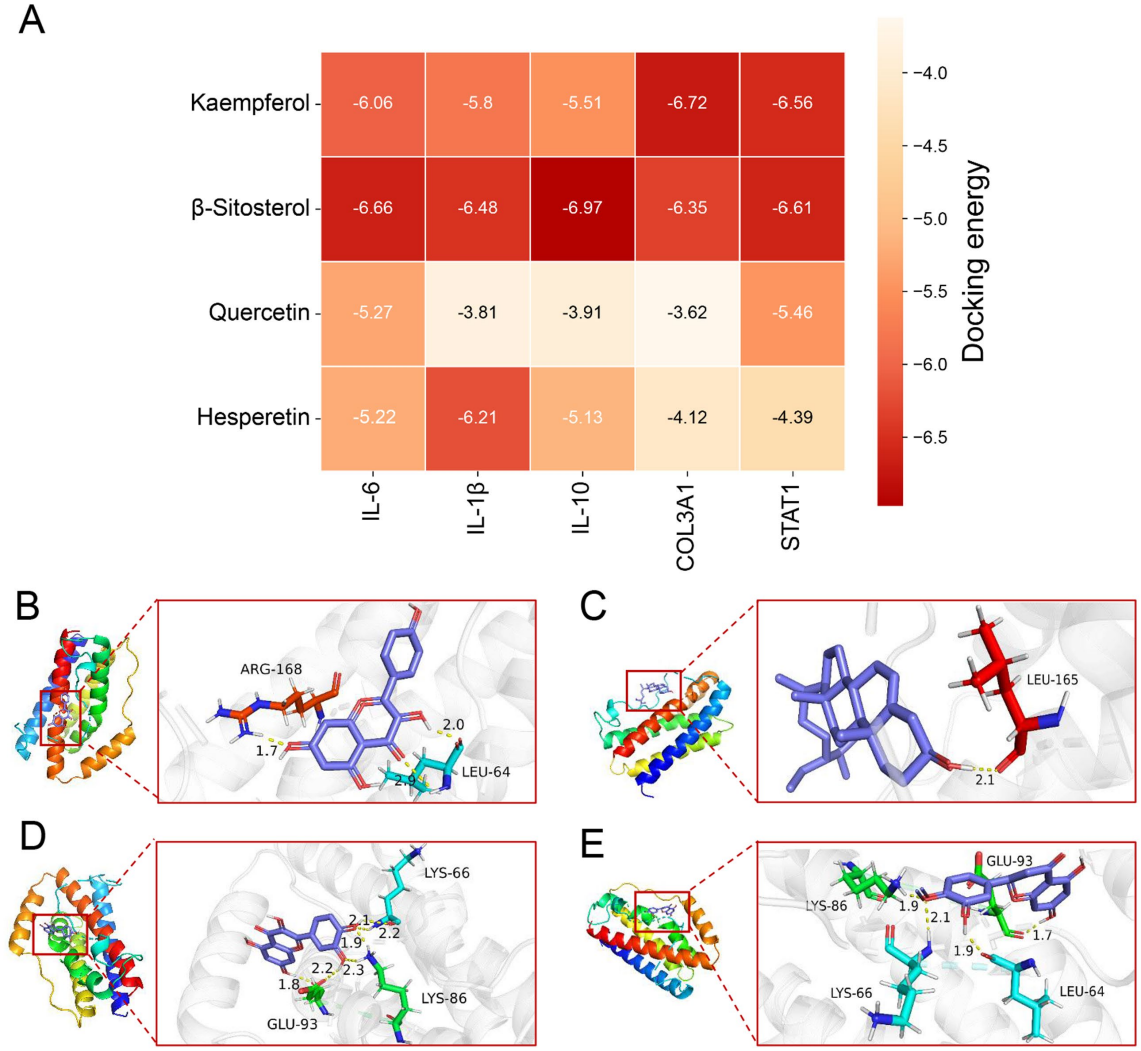
Molecular docking patterns of the primary active components of Wengxian granules and key target proteins. **(A)** The binding energy between the main active components of Wengxian granules and key target proteins. **(B)** Kaempferol and IL-6. **(C)** *β*-Sitosterol and IL-6. **(D)** Quercetin and IL-6. **(E)** Hesperetin and IL-6.

Based on the Wengxian granule–component–common target network, kaempferol, *β*-sitosterol, quercetin, and hesperetin exhibited relatively high degree values and were therefore considered potential key active components. Five core targets identified from the PPI network, namely Interleukin-6 (IL-6), Interleukin-1β (IL-1β), Interleukin-10 (IL-10), collagen alpha-1(III) chain (COL3A1), and signal transducer and activator of transcription 1 (STAT1), were selected for molecular docking analysis with the major active components. The molecular docking results suggested that the active components could stably bind to the core targets, with minimum binding energies ranging from −6.97 to −3.62 kcal/mol (Figure 6A and Supplementary Table S4). Kaempferol and quercetin displayed the favorable binding affinities with all five core targets. Among these interactions, kaempferol with COL3A1 and quercetin with IL-10 showed the strongest binding affinities. These docking results were consistent with the predictions derived from network pharmacology analysis, and representative docking conformations were visualized using PyMOL (Figures 6B–E and Supplementary Tables S1–S4).

## 4 Discussion

Following *Salmonella* invasion in chicks, bacterial LPS activates cellular immune responses. Host antibacterial defense relies on the coordinated actions of macrophages, T cells, and B cells. During the early stage of infection, cellular immunity predominates and is associated with extensive lymphocyte proliferation. When microbial load is high and virulence is strong, pathogens may breach the lymphatic barrier and enter the bloodstream. Under these conditions, circulating immune cells, including lymphocytes and erythrocytes, respond to LPS (38). As an indicator of systemic inflammatory status, changes in white blood cell counts reflect the inflammatory response of the host and provide insight into the anti-inflammatory effects of Wengxian granules. The experimental results demonstrated that in the model group, diarrhea caused by pullorum infection resulted in plasma water loss, increased blood concentration, and a relative increase in cellular blood components, including erythrocytes, which was consistent with the observed alterations in peripheral blood cell profiles.

Immunoglobulins are a class of proteins that share structural and functional characteristics with antibodies. Serum IgG and IgM levels are commonly used as indicators of humoral immune status, whereas sIgA is predominantly produced by the intestinal mucosa (39) and plays a critical role in mucosal immunity. sIgA contributes to intestinal barrier integrity and mediates passive immune protection against enteric pathogens (40). Following *Salmonella* infection, antigenic stimulation induces the production of IgG and IgM in the host. IgG participates in toxin neutralization, whereas IgM plays a major role in limiting bacteremia. To a certain extent, serum immunoglobulin concentrations reflect the disease resistance of the host. In this study, Wengxian granules were administered to prevent and treat pullorum disease in chicks. The results indicated that Wengxian granules suppressed the elevation of IgG and IgM induced by *Salmonella* antigens, contributed to the maintenance of internal homeostasis, enhanced humoral immune balance, and improved disease resistance. After the experimental period, a significant reduction in serum IgG and IgM levels was observed in the model group compared with the control group. Several factors may account for this phenomenon. First, during the self-recovery process in the model group, severe inflammatory responses occurred, and *Salmonella* infection impaired physiological functions, disrupting IgG production. Although partial bacterial clearance occurs after disease resolution, persistent infection leads to subclinical carrier status in some individuals, resulting in weakened or absent antigen responsiveness and subsequent immunosuppression. Second, sustained *Salmonella* infection may have caused damage to lymphoid organs, leading to immune function suppression. Third, extensive bacterial proliferation in organs, such as the liver, may have resulted in substantial antibody consumption during antigen neutralization. During this recovery phase, physiological functions have not yet fully returned to a healthy state, and internal homeostasis remains under regulation.

Endotoxins produced by *Salmonella* can be excreted into the intestinal lumen through the intestinal wall, sensitizing the local intestinal tissues, inducing or exacerbating inflammation, and leading to mucosal swelling, exudation, and epithelial shedding. The intestinal mucosal immune system contains abundant immune cells and cytokines that are essential for maintaining barrier integrity (41). Chinese herbal medicines and their active components have been reported to enhance immune cell activation and regulate lymphocyte proportions, thereby contributing to the establishment of intestinal mucosal immune function and protection of the intestinal barrier. The intestinal immune barrier consists of gut-associated lymphoid tissue and sIgA produced by intestinal plasma cells (42). As a key effector molecule, intestinal sIgA maintains mucosal homeostasis, regulates endogenous microecology, and prevents pathogen adhesion to host epithelial cells by interfering with pathogen–receptor interactions (43). In addition, sIgA influences pathogen virulence and limits further dissemination. The findings of this study indicate that Wengxian granules modulate intestinal immune responses and exert anti-inflammatory effects in the intestinal tract.

In this study, 40 active components in Wengxian granules were screened by network pharmacology using OB and DL values. Flavonoids such as kaempferol, quercetin and hesperetin can significantly improve animal production performance and disease resistance, and improve animal immune function (44–46). Among them, kaempferol has anti-inflammatory and antibacterial effects (47, 48). Quercetin has anti-inflammatory activity, which can reduce the secretion of intestinal water and electrolytes by changing the permeability of abdominal capillaries and intestinal mucosa, so as to achieve the purpose of treating diarrhea (49, 50). Steroidal compounds such as *β*-sitosterol have good anti-inflammatory activity (51). It can be seen that the effective components of Wengxian granules mainly achieve the purpose of improving chicks diarrhea through anti-inflammatory, antibacterial, and improving body immunity.

Through PPI analysis and molecular docking, IL-6, IL-1β, IL-10, COL3A1, and STAT1 were selected from 66 targets as the core targets for the prevention and treatment of avian salmonellosis. IL-6 and IL-1β have a wide range of biological functions in immunity and tissue regeneration (52). Their expression levels rise rapidly after infection, trauma or injury, leading to inflammatory response. IL-10 has a dual role of inhibiting the production of inflammatory factors and promoting the expression of anti-inflammatory proteins (53). COL3A1 is involved in maintaining the normal structure and function of the intestine (54). STAT1 is involved in the immune response of the body (55). Targets such as IL-6, IL-1β, IL-10, COL3A1, and STAT1 are all related to the inflammatory response, indicating that Wengxian granules may treat avian salmonellosis by acting on inflammation-related targets.

The results of GO function and KEGG pathway enrichment analysis showed that the prevention and treatment of avian salmonellosis by Wengxian granules involved multiple biological processes and signaling pathways. According to *p* value, the main signaling pathways such as Toll-like receptor and NOD-like receptor signaling pathways were obtained. Wengxian granules played a role through multiple pathways and multiple targets, so as to prevent and treat avian salmonellosis. The results of network pharmacology prediction provide a reference and direction for our future in-depth research.

## 5 Conclusion

In summary, this study demonstrated that Wengxian granules exerted protective effects against avian *Salmonella* infection by modulating both cell-mediated and antibody-mediated immune responses. Additionally, the active components and potential therapeutic targets of Wengxian granules were systematically identified, and robust interactions between key targets and major active compounds were confirmed using network pharmacology analysis and molecular docking. Collectively, these findings provide experimental and theoretical support for the application of Wengxian granules as an effective strategy for the prevention and treatment of avian salmonellosis.

## Data Availability

The original contributions presented in the study are included in the article/Supplementary material, further inquiries can be directed to the corresponding author.
