# Supplementary material for: A systems pharmacology-based in vivo study elucidating the mechanism of Wengxian granules against avian salmonellosis
Source: Front Vet Sci. 2026 Mar 18;13:1790708. doi: 10.3389/fvets.2026.1790708 (PMC13038521; doi:10.3389/fvets.2026.1790708)
Supplement: Supplementary file 1 [file Data_Sheet_1.docx]

**Supporting Information for**

**A systems pharmacology-based *in* *vivo* study elucidating the mechanism of Wengxian granules against avian salmonellosis**

Siting Pu^1#^, Lihui Zhang^2#^, Hongzao Yang^1^, Guangming Wang^1^, Tingting Liu^1^, Hongwei Chen^1^, Wei Wei^1*^

1. College of Veterinary Medicine, Southwest University, 2 Tiansheng Rd, Beibei District, Chongqing, 400715, China

2. School of Pharmaceutical Sciences, Tongren Polytechnic University, 2 Ziyou Road, Bijiang District, Guizhou，554300, China

* Corresponding Author: College of Veterinary Medicine, Southwest University, 2 Tiansheng Rd, Beibei District, Chongqing, 400715, China; E-mail: wei2023@swu.edu.cn

Number of pages (11), figures (4), and tables (4) in the SI.

**Table S1.** Pathological scoring criteria of cecum HE staining.

| Score | Tissue structure changes | Leukocyte changes in lamina propria | Crypt changes | Erosion and ulceration |
| --- | --- | --- | --- | --- |
| 1 | none | none | none | none |
| 2 | ≤5% | ≤5% | ≤5% | ≤5% |
| 3 | 6-50% | 6-50% | 6-50% | 6-50% |
| 4 | >50% | >50% | >50% | >50% |

**Table S2.** Main active ingredients of Wengxian granules.

| Mol ID | Molecule Name | Oral Bioavailability (%) | Drug-likeness | Source |
| --- | --- | --- | --- | --- |
| MOL000422 | Kaempferol | 41.88 | 0.24 | *Portulaca oleracea*  *Paeoniae Radix* Alba |
| MOL000358 | β-sitosterol | 36.91 | 0.75 | *Portulaca oleracea*  *Paeoniae Radix* Alba *Pulsatillae radix‌* |
| MOL000098 | Quercetin | 46.43 | 0.28 | *Portulaca oleracea* |
| MOL005100 | Hesperetin | 47.74 | 0.27 | *Portulaca oleracea*  *Pericarpium Citri* Reticulatae |
| MOL000006 | Luteolin | 36.16 | 0.25 | *Portulaca oleracea* |
| MOL000173 | Wogonin | 30.68 | 0.23 | *Atractylodis macrocephalae* Rhizoma‌ |
| MOL001439 | Arachidonic acid-d5 | 45.57 | 0.2 | *Portulaca oleracea* |
| MOL000354 | Isorhamnetin | 49.6 | 0.31 | *Pulsatillae radix‌* |
| MOL000211 | Betulinic acid | 55.38 | 0.78 | *Pulsatillae radix‌* *Paeoniae Radix* Alba |
| MOL005980 | Neohesperidin | 57.44 | 0.27 | *Magnolia officinalis* |
| MOL004328 | naringenin | 59.29 | 0.21 | *Pericarpium Citri* Reticulatae |
| MOL001910 | 11alpha,12alpha-epoxy-3beta-23-dihydroxy-30-norolean-20-en-28,12beta-olide | 64.77 | 0.38 | *Paeoniae Radix* Alba |
| MOL000359 | sitosterol | 36.91 | 0.75 | *Paeoniae Radix* Alba  *Pericarpium Citri* Reticulatae |
| MOL000179 | 2-Hydroxyisoxypropyl-3-hydroxy-7-isopentene-2,3-dihydrobenzofuran-5-carboxylic | 45.2 | 0.2 | *Atractylodis macrocephalae* Rhizoma‌ |
| MOL000449 | Stigmasterol | 43.83 | 0.76 | *Pulsatillae radix‌* |
| MOL005970 | Eucalyptol | 60.62 | 0.27 | *Magnolia officinalis* |
| MOL001979 | LAN | 42.12 | 0.75 | *Pulsatillae radix‌* |
| MOL001924 | paeoniflorin | 53.87 | 0.79 | *Paeoniae Radix* Alba |
| MOL001971 | Pulchinenoside C_qt | 37.79 | 0.76 | *Pulsatillae radix‌* |
| MOL000184 | NSC63551 | 39.25 | 0.76 | *Atractylodis macrocephalae* Rhizoma‌ |
| MOL005828 | nobiletin | 61.67 | 0.52 | *Pericarpium Citri* Reticulatae |
| MOL000092 | daucosterin_qt | 36.91 | 0.76 | *Atractylodis macrocephalae* Rhizoma‌ |
| MOL001930 | benzoyl paeoniflorin | 31.27 | 0.75 | *Paeoniae Radix* Alba |
| MOL000186 | Stigmasterol 3-O-beta-D-glucopyranoside_qt | 43.83 | 0.76 | *Atractylodis macrocephalae* Rhizoma‌ |
| MOL001978 | Aureusidin | 53.42 | 0.24 | *Pulsatillae radix‌* |
| MOL001973 | Sitosteryl acetate | 40.39 | 0.85 | *Pulsatillae radix‌* |
| MOL003578 | Cycloartenol | 38.69 | 0.78 | *Portulaca oleracea* |
| MOL002773 | beta-carotene | 37.18 | 0.58 | *Portulaca oleracea* |
| MOL000188 | 3β-acetoxyatractylone | 40.57 | 0.22 | *Atractylodis macrocephalae* Rhizoma‌ |
| MOL005815 | Citromitin | 86.9 | 0.51 | *Pericarpium Citri* Reticulatae |
| MOL000569 | digallate | 61.85 | 0.26 | *Galla chinensis* |
| MOL001985 | ZINC01615307 | 56.38 | 0.87 | *Pulsatillae radix‌* |
| MOL001984 | 3beta,23-Dihydroxy-lup-20(29)-ene-28-O-alpha-L-rhamnopyranosyl-(1-4)-beta-D-glucopyranosyl(1-6)-beta-D-glucopyranoside_qt | 37.59 | 0.79 | *Pulsatillae radix‌* |
| MOL001919 | (3S,5R,8R,9R,10S,14S)-3,17-dihydroxy-4,4,8,10,14-pentamethyl-2,3,5,6,7,9-hexahydro-1H-cyclopenta[a]phenanthrene-15,16-dione | 43.56 | 0.53 | *Paeoniae Radix* Alba |
| MOL006657 | isobetanidin | 59.73 | 0.52 | *Portulaca oleracea* |
| MOL006662 | isobetanin_qt | 30.16 | 0.52 | *Portulaca oleracea* |
| MOL000492 | (+)-catechin | 54.83 | 0.24 | *Paeoniae Radix* Alba |
| MOL001987 | β-sitosterol | 33.94 | 0.7 | *Pulsatillae radix‌* |
| MOL001918 | paeoniflorgenone | 87.59 | 0.37 | *Paeoniae Radix* Alba |
| MOL000085 | beta-daucosterol_qt | 36.91 | 0.75 | *Atractylodis macrocephalae* Rhizoma‌ |

**Table S3**. The top 20 key targets in order of degree.

| Gene name | Degree | Betweenness Centrality | Closeness Centrality | Topological Coefficient |
| --- | --- | --- | --- | --- |
| IL-6 | 28 | 0.16430 | 0.77551 | 0.36508 |
| IL-1β | 27 | 0.15303 | 0.74510 | 0.37037 |
| IL-10 | 21 | 0.06630 | 0.66667 | 0.50000 |
| COL3A1 | 21 | 0.07282 | 0.66667 | 0.48571 |
| STAT1 | 19 | 0.05891 | 0.63333 | 0.53216 |
| IFNG | 18 | 0.03768 | 0.62295 | 0.56209 |
| ALB | 18 | 0.03361 | 0.63333 | 0.56209 |
| MMP9 | 18 | 0.06037 | 0.62295 | 0.54902 |
| EGFR | 15 | 0.06707 | 0.59375 | 0.57143 |
| IL-4 | 15 | 0.02260 | 0.59375 | 0.63810 |
| CASP8 | 15 | 0.01678 | 0.58462 | 0.63810 |
| CASP1 | 12 | 0.00560 | 0.53521 | 0.72727 |
| JAK1 | 11 | 0.03960 | 0.55882 | 0.67273 |
| JUN | 9 | 0.00014 | 0.53521 | 0.97222 |
| IL-2 | 9 | 0.00055 | 0.51351 | 0.94444 |
| MMP2 | 8 | 0.01139 | 0.52055 | 0.78571 |
| TNFRSF1A | 8 | 0.00569 | 0.50667 | 0.67857 |
| PLAU | 8 | 0.00047 | 0.51351 | 0.92857 |
| MMP7 | 8 | 0.00253 | 0.52055 | 0.85714 |
| ZAP70 | 7 | 0.03662 | 0.49351 | 0.66667 |

**Table S4**. Binding energy of main active ingredients in Wengxian granules and key target proteins.

| Molecule name | Binding protein | Docking score (kcal/mol) | Binding site |
| --- | --- | --- | --- |
| Kaempferol | IL-6 | -6.06 | AGR168, LEU64 |
| Kaempferol | IL-1β | -5.8 | MET20, VAL41, LYS63 |
| Kaempferol | IL-10 | -5.51 | ARG102, LYS99, GLU74, ASN92, VAL91 |
| Kaempferol | COL3A1 | -6.72 | ARG213, LEU138, ARG213, ALA215, VAL216 |
| Kaempferol | STAT1 | -6.56 | GLN41, TRP43, LEU109, TYR106, ARG113, GLN3 |
| β-sitosterol | IL-6 | -6.66 | LEU165 |
| β-sitosterol | IL-1β | -6.48 | LYS27 |
| β-sitosterol | IL-10 | -6.97 | LEU47, LEU48 |
| β-sitosterol | COL3A1 | -6.35 | LYS186 |
| β-sitosterol | STAT1 | -6.61 | ASN1357 |
| Quercetin | IL-6 | -5.27 | LYS66, LYS86, GLU93 |
| Quercetin | IL-1β | -3.81 | LYS63, VAL41, GLU64, LYS65 |
| Quercetin | IL-10 | -3.91 | ASN92, ARG102, GRU74 |
| Quercetin | COL3A1 | -3.62 | ALA134, VAL216, ARG137, ALA215, SER140 |
| Quercetin | STAT1 | -5.46 | THR1387, ASP168, GLN167 |
| Hesperetin | IL-6 | -5.22 | LYS86, LYS66, LEU64, GLU93 |
| Hesperetin | IL-1β | -6.21 | ASN7, GLU64, LYS65, TYR90, PRO87 |
| Hesperetin | IL-10 | -5.13 | GLU74, ARG102 |
| Hesperetin | COL3A1 | -4.12 | ALA215, LEU138 |
| Hesperetin | STAT1 | -4.39 | ASN1357, ASN1355, GLN275, GLU157 |

**Supplementary Figures**


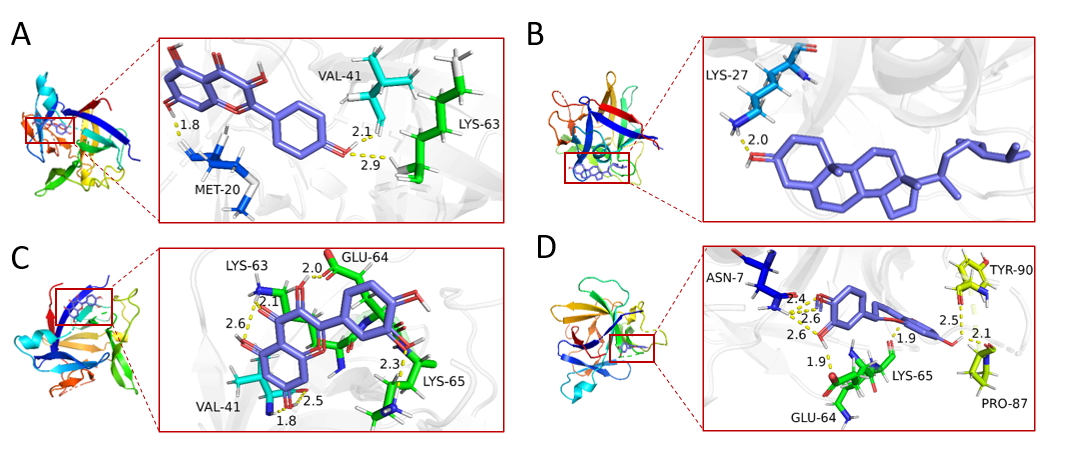


**Figure S1.** **Molecular docking patterns of the primary active components of Wengxian granules and IL1-β.** (A) Kaempferol and IL-1β. (B) β-sitosterol and IL-1β. (C) Quercetin and IL-1β. (D) Hesperetin and IL-1β.


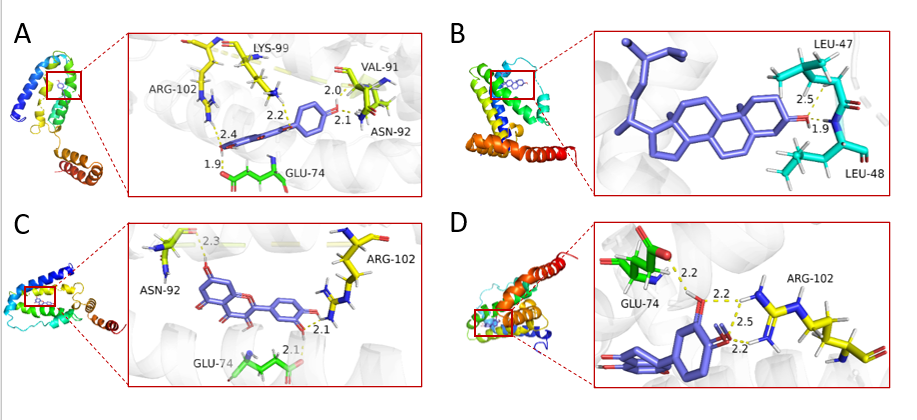


**Figure S2.** **Molecular docking patterns of the primary active components of Wengxian granules and IL-10.** (A) Kaempferol and IL-10. (B) β-sitosterol and IL-10. (C) Quercetin and IL-10. (D) Hesperetin and IL-10.


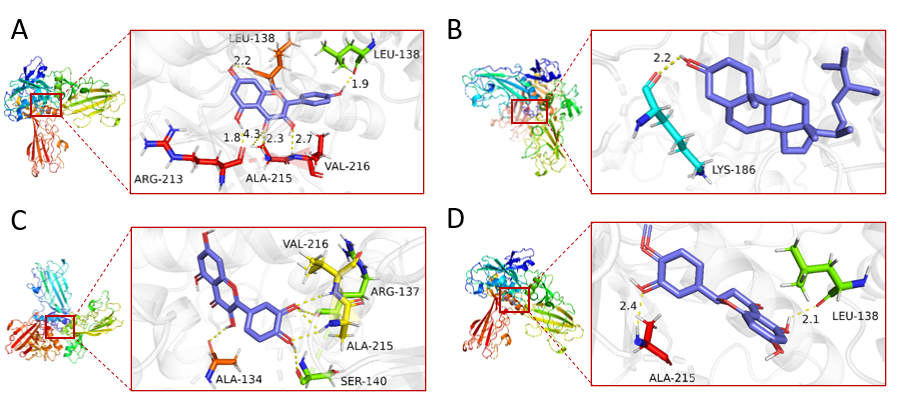


**Figure S3.** **Molecular docking patterns of the primary active components of Wengxian granules and COL3A1.** (A) Kaempferol and COL3A1. (B) β-sitosterol and COL3A1. (C) Quercetin and COL3A1. (D) Hesperetin and COL3A1.


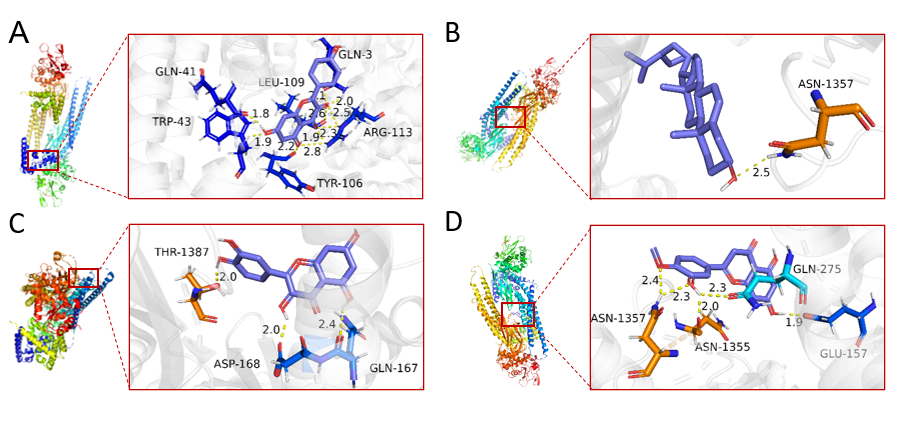


**Figure S4.** **Molecular docking patterns of the primary active components of Wengxian granules and STAT1.** (A) Kaempferol and STAT1. (B) β-sitosterol and STAT1. (C) Quercetin and STAT1. (D) Hesperetin and STAT1.
